# Prognostic values of the prognostic nutritional index, geriatric nutritional risk index, and systemic inflammatory indexes in patients with stage IIB–III cervical cancer receiving radiotherapy

**DOI:** 10.3389/fnut.2023.1000326

**Published:** 2023-03-02

**Authors:** Hong-Bing Wang, Xin-Tian Xu, Meng-Xing Tian, Chen-Chen Ding, Jing Tang, Yu Qian, Xin Jin

**Affiliations:** ^1^Department of Gynecology and Oncology, Hubei Cancer Hospital, Tongji Medical College, Huazhong University of Science and Technology, Wuhan, Hubei, China; ^2^Department of Pharmacy, Hubei Cancer Hospital, Tongji Medical College, Huazhong University of Science and Technology, Wuhan, Hubei, China; ^3^Department of Clinical Nutrition, Hubei Cancer Hospital, Tongji Medical College, Huazhong University of Science and Technology, Wuhan, Hubei, China; ^4^Department of Lymphoma Medicine, Breast Cancer and Soft Tissue Tumor Medicine, Hubei Cancer Hospital, Tongji Medical College, Huazhong University of Science and Technology, Wuhan, Hubei, China; ^5^Department of Thoracic Oncology, Hubei Cancer Hospital, Tongji Medical College, Huazhong University of Science and Technology, Wuhan, Hubei, China

**Keywords:** prognostic nutritional index, geriatric nutritional risk index, systemic inflammatory indexes, cervical cancer, overall survival

## Abstract

**Background:**

Growing evidence suggests that nutritional status and inflammation are associated with survival in various cancers. This study aimed to evaluate the prognostic value of the prognostic nutritional index (PNI), geriatric nutritional risk index (GNRI), and systemic inflammatory indexes (neutrophil/lymphocyte ratio [NLR], monocyte/lymphocyte ratio [MLR], and platelet/lymphocyte ratio [PLR]) in patients with stage IIB–III cervical cancer receiving radiotherapy.

**Results:**

The ideal cutoff values for the PNI, GNRI, NLR, MLR, and PLR were 48.3, 97.04, 2.8, 0.41, and 186.67, respectively. Low PNI and GNRI scores were associated with poor OS and PFS. High NLR, MLR, and PLR also predicted inferior 5-year OS and PFS rates in patients with stage IIB–III cervical cancer. Multivariate Cox regression analysis identified tumor size, histological type, stage, number of metastatic lymph nodes, PNI, GNRI, NLR, PLR, and MLR as significant prognostic factors for OS and PFS.

**Conclusions:**

The current findings suggest that the PNI, GNRI, NLR, PLR, and MLR are essential parameters for predicting prognosis in patients with stage IIB–III cervical cancer receiving radiotherapy.

## 1. Introduction

Although largely preventable, cervical cancer is the fourth most common cancer in women in the USA and worldwide (1). In 2020, approximately 604,000 new cases and 341,000 deaths were reported due to cervical cancer (2). Unfortunately, more than two-thirds of women with cervical cancer are diagnosed at advanced stages in developing countries (3, 4). In patients with locally advanced cervical cancer, survival is worse, and the recurrence rate is higher than that in those with early-stage cancer. The 5-year survival rate ranges from 31 to 55% in patients with locally advanced cervical cancer undergoing optimal treatment such as chemoradiotherapy (5). Staging, nodal involvement, and human papillomavirus infection affect local control and survival and have been used to predict treatment outcomes in patients with cervical cancer (6–8). However, the existing staging systems and other prognostic factors are not perfect to predict prognosis (9). For example, although some patients have the same International Federation of Gynecology and Obstetrics (FIGO) stage, their prognosis is disparate because of their different pathological types (10, 11). In addition, nutrition status is recognized as a critical determinant of quality of life in patients with cancer (12). It is inherently inaccurate to predict the prognosis using only the existing system if the patient is malnourished. Accordingly, several novel prognostic parameters, a model with the existing system, and novel markers are required to predict life expectancy.

Nutritional status is recognized as a critical determinant of quality of life in patients with cancer (12). Several studies have verified that malnutrition, sarcopenia, and cancer cachexia are associated with higher rates of post-treatment complications, lower rates of clinical response, longer hospital stays, and shorter survival times (13–17). In recent studies, several parameters, including nutritional and inflammatory indicators, have been shown to predict the prognosis of different tumors (18–20). PNI, an easily obtained index for evaluating nutritional status by calculating serum albumin levels and absolute lymphocyte counts, was first introduced to predict operative risk in gastrointestinal surgery (21). Several retrospective studies have indicated that the prognostic nutritional index (PNI) is associated with clinical outcomes in many types of cancer (22, 23). The geriatric nutritional risk index (GNRI) is calculated using serum albumin levels and ideal body weight. A low GNRI has also been verified as an independent prognostic factor affecting overall survival (OS) in patients with cancer (24).

Many studies have demonstrated the value of inflammatory cells in the blood and systemic inflammatory responses in the prognosis of patients with various types of tumors (25). A series of systemic inflammatory indexes, such as the neutrophil/lymphocyte ratio (NLR), platelet/lymphocyte ratio (PLR), and monocyte/lymphocyte ratio (MLR), can be obtained in an easily available and inexpensive manner. The prognostic roles of NLR, PLR, and MLR have been verified in lung cancer, colorectal cancer, and hepatocellular carcinoma (26–28). For patients with operable cervical cancer, the prognostic value of NLR, PLR, and MLR has been investigated after surgery (29–32). Some studies have also reported the prognostic value of systemic inflammatory indexes in patients with non-surgical cervical cancer. One study reported that NLR and MLR predicted poor OS in patients with cervical cancer; however, only patients with stage IIB cancer were analyzed (33). A retrospective study found that pretreatment NLR and PNI were significant predictors of prognosis in patients with cervical cancer treated with concurrent chemoradiotherapy (34). However, many patients with stage I and IV disease were also included in the aforementioned study, and the prognosis of these patients was significantly different from that of patients with stage II–III disease. Moreover, survival curves and log-rank tests for different PNI/NLR/PLR values were not performed in Haraga et al.'s research. To date, there have been no reports on the impact of PNI, GNRI, NLR, PLR, and MLR on predicting survival time in patients with stage IIB–III disease undergoing radiotherapy (RT). Therefore, this study aimed to retrospectively analyze whether these factors are significantly associated with the prognosis of patients with stage IIB–III disease treated with RT.

## 2. Methods and materials

### 2.1. Study population

Data from patients with cervical cancer who underwent RT were collected at the Hubei Cancer Hospital of Huazhong University of Science and Technology. A total of 178 patients were enrolled in this retrospective study from September 2013 to September 2015. Patients with incomplete medical records were excluded. As this was a retrospective study and the data were anonymous, the requirement for informed consent was waived. This study was approved by the Ethics Committee of Hubei Cancer Hospital of Huazhong University of Science and Technology (LLHBCH2021YN-049).

### 2.2. Data collection

The demographic characteristics, clinical characteristics, and laboratory results of the 178 patients were obtained from medical records. Data on age, body weight, tumor size, tumor stage, serum levels of squamous cell carcinoma (SCC) antigen, number of metastatic lymph nodes, serum albumin, and platelet, neutrophil, lymphocyte, and monocyte counts were collected. The International Federation of Gynecology and Obstetrics (FIGO) 2009 clinical staging system was used for tumor staging. Blood samples were collected before RT. Routine blood tests were performed using the Sysmex XN-9000 Hematology System (Sysmex Corporation, Shanghai, China). Biochemical tests were performed using an ADVIA 2400 Clinical Chemistry System (Siemens Healthineers, Erlangen, Germany). Serum SCC antigen tests were performed using a Cobas e 801 analytical unit (Roche Diagnostics International AG, Rotkreuz, Switzerland) and body weight was measured before treatment. The PNI and GNRI were calculated using the following formulas: PNI = serum albumin (g/L) + 5 × absolute lymphocyte count (10^9^/L) and GNRI = [14.89 × serum albumin level (g/dL)] + [41.7 × actual body weight/ideal body weight]. NLR, PLR, and MLR were calculated as neutrophil/lymphocyte, platelet/lymphocyte, and monocyte/lymphocyte ratios, respectively.

### 2.3. RT procedures

Patients with cervical cancer (FIGO stages IIB–III) were treated with RT. If possible, after the initiation of RT, cisplatin at a dose of 40 mg/m^2^ on the body surface was also administered. A total of 105 patients underwent intensity-modulated RT (IMRT). The gross, clinical, and planned tumor volumes for patients who received IMRT were defined according to the Radiation Therapy Oncology Group guidelines. The prescribed dose was 45.0–50.4 Gy. IMRT was delivered at 1.8 Gy per fraction once daily for 5 days per week. A total of 73 patients underwent conventional RT (CRT). CRT was planned using the Eclipse Planning System and was conducted using a Varian 23EX. Conventional RT was delivered using anterior and posterior opposing techniques at a dose of 45.0–50.4 Gy (1.8 Gy per day, 5 days per week). All patients underwent a high dose of ^192^Ir brachytherapy after whole-pelvic irradiation at a dose of up to 36 Gy.

### 2.4. Follow-up strategy

Patients were followed up *via* outpatient examinations or telephone calls. The deadline for follow up was December 2019. OS was defined as the time from the start of RT to the date of death or last follow up. Progression-free survival (PFS) was defined as the initiation of RT, occurrence of tumor progression, death from any cause, or the last follow up.

### 2.5. Statistical analysis

Receiver operating characteristic (ROC) curves were used to determine the optimal PNI, GNRI, PLR, MLR, and NLR cutoff points using MedCalc (MedCalc Software Ltd., Belgium). R software version 4.1.3 (The R Foundation, Vienna, Austria) was used for statistical analysis. For the baseline characteristics of the patients, means and standard deviations are used to express continuous variables. Numbers and percentages are used to express categorical variables. Descriptive analysis using the chi-square test or Fisher's exact test was performed to compare differences between the two groups. Survival curves were calculated using the Kaplan–Meier method, and the log-rank test was used for comparison. Univariate and multivariate analyses were performed for each marker using the Cox proportional hazards model. Variables that were significant in the univariate analysis with *P*-values < 0.20 were included in multivariate analysis. We applied the nomogram in this study and visualized the prognostic strengths of different factors in predicting OS.

## 3. Results

### 3.1. Patient characteristics

**Figure 1:**
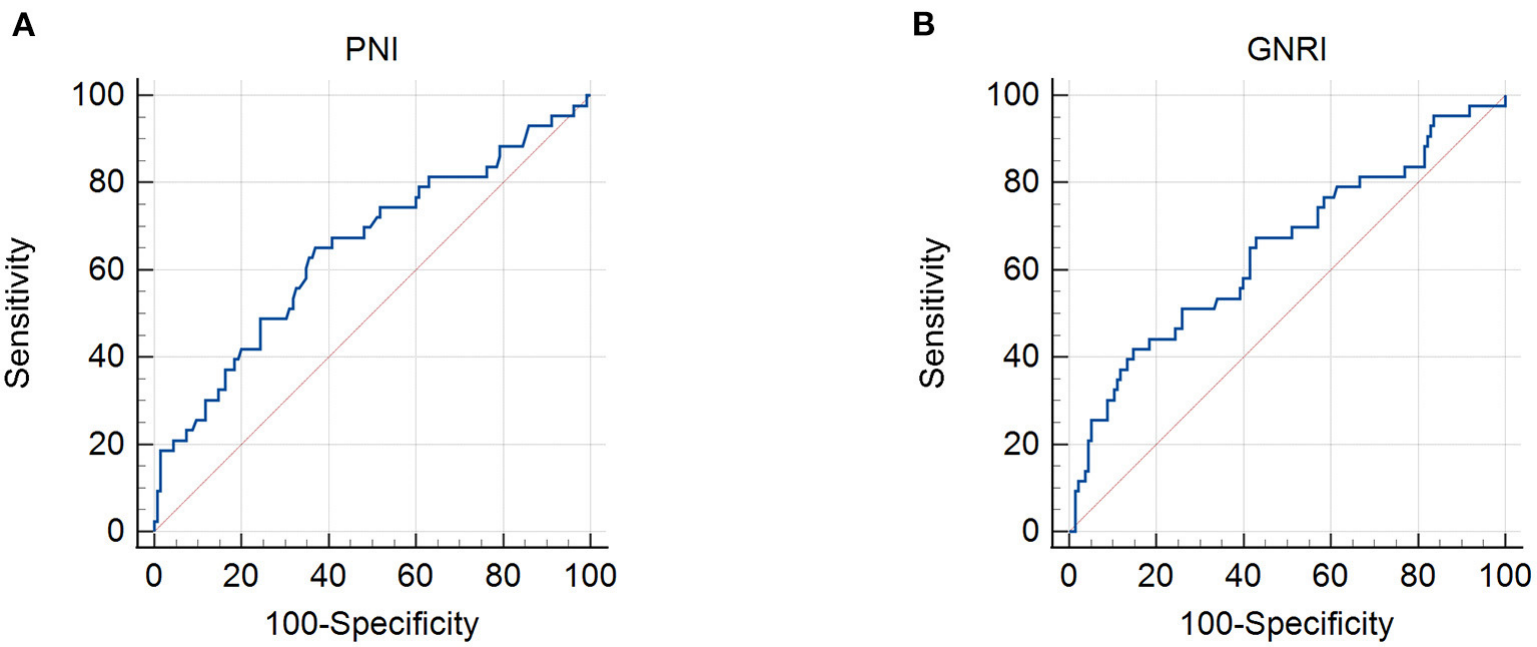
Receiver operating characteristic curves for the use of nutritional indicators to predict life expectancy in patients with stage IIB–III cervical cancer receiving radiotherapy based on: **(A)** prognostic nutritional index (PNI) and **(B)** geriatric nutritional risk index (GNRI). Cut-off points with the highest combined sensitivity and specificity were used.

**Figure 2:**
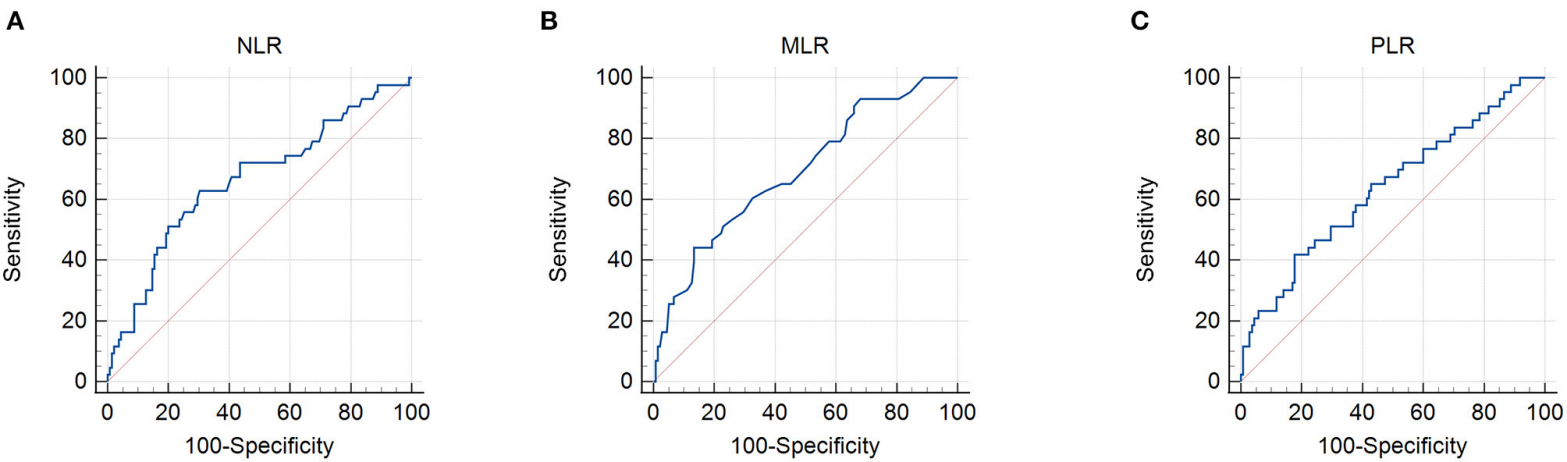
Receiver operating characteristic curves for the use of inflammatory indicators to predict life expectancy in patients with stage IIB–III cervical cancer receiving radiotherapy based on: **(A)** neutrophil/lymphocyte ratio (NLR), **(B)** monocyte/lymphocyte ratio (MLR), and **(C)** platelet/lymphocyte ratio (PLR). Cut-off points with the highest combined sensitivity and specificity were used.

The patient characteristics are presented in Table 1. A total of 178 patients with cervical cancer were enrolled in this retrospective study. The mean age was 53.85. Thirty patients out of 178 (16.9%) had more than two positive metastatic lymph nodes. Ninety-four patients (52.8%) had stage II tumors and 84 (47.2%) had stage III tumors, according to the FIGO 2009 clinical staging system. The mean body mass index (BMI) was 23.19 ± 2.88 kg/m^2^ with 3.4% of patients being underweight. By setting survival status as an endpoint, ROC curves were used to determine the cutoff values. The cutoff values for the PNI, GNRI, NLR, MLR, and PLR were 48.3, 97.04, 2.8, 0.41, and 186.67, respectively (Figures 1, 2). The mean PNI, GNRI, NLR, MLR, and PLR were 49.37, 102.74, 2.77, 0.3, and 159.26, respectively. Low PNI and GNRI scores were observed in 78 (43.8%) and 37 (20.8%) patients, respectively. Low NLR, MLR, and PLR values were observed in 110 (61.8%), 141 (79.2%), and 136 (76.4%) patients, respectively (Table 1).

**Table 1:** The baseline characteristics of 178 patients with cervical cancer.

**Patients features**	**PNI ≤ 48.3**	**PNI > 48.3**	***P*-value**	**GNRI ≤ 97.04**	**GNRI > 97.04**	***P*-value**	**NLR ≤ 2.8**	**NLR> 2.8**	***P*-value**	**MLR ≤ 0.41**	**MLR > 0.41**	***P*-value**	**PLR ≤ 186.67**	**PLR> 186.67**	***P*-value**
No. of patients	78	100		37	141		110	68		141	37		136	42	
Age [mean (SD)]	52.46 (9.06)	54.93 (9.59)		52.57 (9.22)	54.18 (9.47)		54.81 (9.02)	52.29 (9.90)		54.52 (8.89)	51.27 (10.97)		55.55 (9.22)	48.33 (7.88)	
≤ 55 [n, (%)]	54 (69.23)	47 (47.00)	0.005	26 (70.27)	75 (53.19)	0.093	58 (52.73)	43 (63.24)	0.223	76 (53.90)	25 (67.57)	0.191	66 (48.53)	35 (83.33)	< 0.001
>55 [n, (%)]	24 (30.77)	53 (53.00)		11 (29.73)	66 (46.81)		52 (47.27)	25 (36.76)		65 (46.10)	12 (32.43)		70 (51.47)	7 (16.67)	
**No. of metastatic lymph nodes**															
≤ 2 [*n*, (%)]	62 (79.49)	86 (86.00)	0.342	27 (72.97)	121 (85.82)	0.107	92 (83.64)	56 (82.35)	0.987	121 (85.82)	27 (72.97)	0.107	115 (84.56)	33 (78.57)	0.503
>2 [*n*, (%)]	16 (20.51)	14 (14.00)		10 (27.03)	20 (14.18)		18 (16.36)	12 (17.65)		20 (14.18)	10 (27.03)		21 (15.44)	9 (21.43)	
**Size of metastatic lymph nodes**															
≤ 1 cm [*n*, (%)]	10 (12.82)	11 (11.00)	0.411	2 (5.41)	19 (13.48)	0.024	11 (10.00)	10 (14.71)	0.409	14 (9.93)	7 (18.92)	0.072	13 (9.56)	8 (19.05)	0.035
>1 cm [*n*, (%)]	24 (30.77)	23 (23.00)		16 (43.24)	31 (21.99)		27 (24.55)	20 (29.41)		34 (24.11)	13 (35.14)		32 (23.53)	15 (35.71)	
No metastatic lymph nodes [*n*, (%)]	44 (56.41)	66 (66.00)		19 (51.35)	91 (64.54)		72 (65.45)	38 (55.88)		93 (65.96)	17 (45.95)		91 (66.91)	19 (45.24)	
**Size of tumor**															
≤ 4 cm [*n*, (%)]	31 (39.74)	57 (57.00)	0.033	14 (37.84)	74 (52.48)	0.161	64 (58.18)	24 (35.29)	0.005	74 (52.48)	14 (37.84)	0.161	73 (53.68)	15 (35.71)	0.063
>4 cm [*n*, (%)]	47 (60.26)	43 (43.00)		23 (62.16)	67 (47.52)		46 (41.82)	44 (64.71)		67 (47.52)	23 (62.16)		63 (46.32)	27 (64.29)	
**Type to radiotherapy**															
IMRT [*n*, (%)]	42 (53.85)	63 (63.00)	0.281	19 (51.35)	86 (60.99)	0.382	70 (63.64)	35 (51.47)	0.148	80 (56.74)	25 (67.57)	0.315	78 (57.35)	27 (64.29)	0.536
RT [*n*, (%)]	36 (46.15)	37 (37.00)		18 (48.65)	55 (39.01)		40 (36.36)	33 (48.53)		61 (43.26)	12 (32.43)		58 (42.65)	15 (35.71)	
**Pathology**															
Squamous cell carcinoma [*n*, (%)]	69 (88.46)	93 (93.00)	0.432	35 (94.59)	127 (90.07)	0.594	101 (91.82)	61 (89.71)	0.834	128 (90.78)	34 (91.89)	1.000	123 (90.44)	39 (92.86)	0.865
Adenocarcinoma [*n*, (%)]	9 (11.54)	7 (7.00)		2 (5.41)	14 (9.93)		9 (8.18)	7 (10.29)		13 (9.22)	3 (8.11)		13 (9.56)	3 (7.14)	
**FIGO stage**															
II [*n*, (%)]	36 (46.15)	58 (58.00)	0.156	15 (40.54)	79 (56.03)	0.135	63 (57.27)	31 (45.59)	0.173	81 (57.45)	13 (35.14)	0.025	76 (55.88)	18 (42.86)	0.193
III [*n*, (%)]	42 (53.85)	42 (42.00)		22 (59.46)	62 (43.97)		47 (42.73)	37 (54.41)		60 (42.55)	24 (64.86)		60 (44.12)	24 (57.14)	
**SCC antigen**															
≤ 1.5 [*n*, (%)]	19 (24.36)	28 (28.00)	0.707	7 (18.92)	40 (28.37)	0.342	32 (29.09)	15 (22.06)	0.39	41 (29.08)	6 (16.22)	0.171	39 (28.68)	8 (19.05)	0.3
> 1.5 [*n*, (%)]	59 (75.64)	72 (72.00)		30 (81.08)	101 (71.63)		78 (70.91)	53 (77.94)		100 (70.92)	31 (83.78)		97 (71.32)	34 (80.95)	
**Chemoradiotherapy**															
No [*n*, (%)]	2 (2.56)	5 (5.00)	0.659	1 (2.70)	6 (4.26)	1	3 (2.73)	4 (5.88)	0.512	5 (3.55)	2 (5.41)	0.966	6 (4.41)	1 (2.38)	0.89
Yes [*n*, (%)]	76 (97.44)	95 (95.00)		36 (97.30)	135 (95.74)		107 (97.27)	64 (94.12)		136 (96.45)	35 (94.59)		130 (95.59)	41 (97.62)	
Height (cm) [mean (SD)]	157.06 (4.80)	157.22 (4.06)	0.815	156.49 (4.90)	157.33 (4.24)	0.301	156.88 (4.38)	157.59 (4.39)	0.298	157.11 (4.49)	157.32 (4.03)	0.789	156.88 (4.30)	158.02 (4.59)	0.141
Bodyweight (Kg) [mean (SD)]	56.06 (8.01)	58.33 (7.97)	0.062	50.47 (6.00)	59.13 (7.54)	< 0.001	58.24 (8.00)	55.87 (7.95)	0.056	57.43 (7.82)	56.96 (8.96)	0.753	57.86 (7.84)	55.62 (8.54)	0.115
ALB (g/L) [mean (SD)]	38.29 (3.30)	43.58 (2.68)	< 0.001	36.41 (3.58)	42.54 (2.94)	< 0.001	41.84 (3.48)	40.33 (4.51)	0.013	41.79 (3.50)	39.24 (4.91)	< 0.001	41.62 (4.14)	40.12 (3.06)	0.031

IMRT, Intensity-modulated radiotherapy; SCC, Squamous cell carcinoma; PNI, Prognostic nutritional index; GNRI, Geriatric nutritional risk index; NLR, Neutrophil/lymphocyte ratio; MLR, Monocyte/lymphocyte ratio; PLR, Platelet/lymphocyte ratio.

### 3.2. Prognostic value of PNI and GNRI

**Figure 3:**
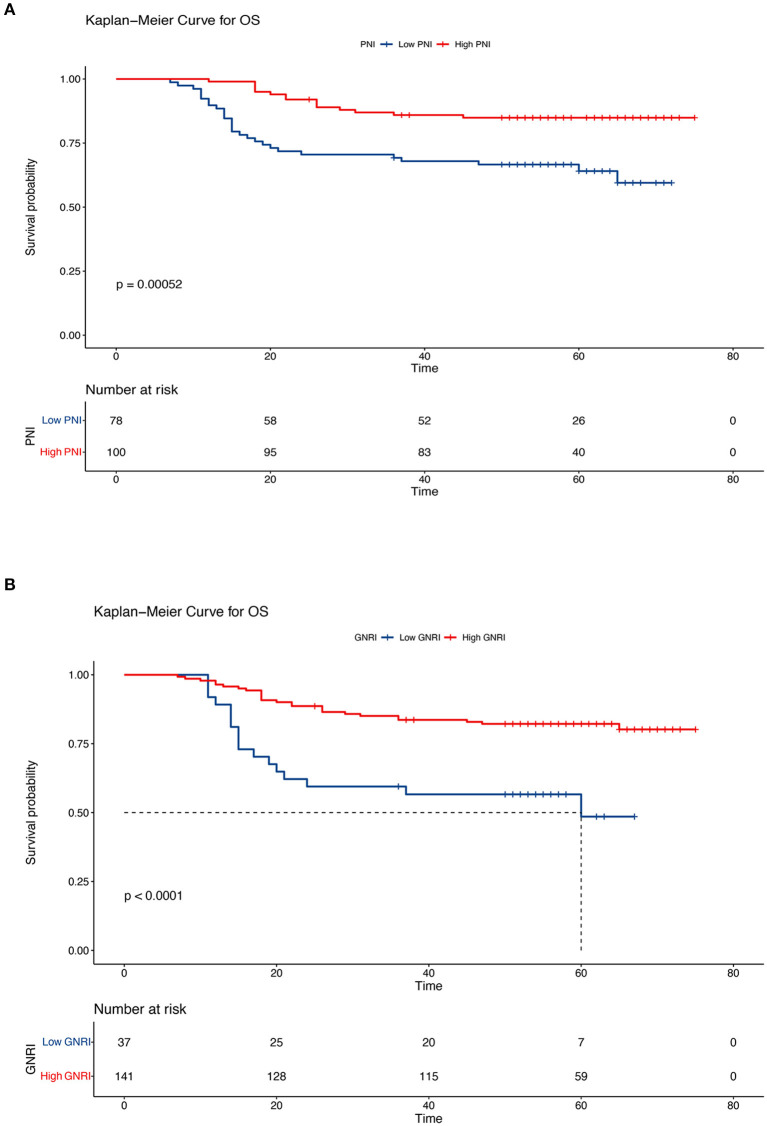
Kaplan–Meier curves of overall survival according to the nutritional indicators. **(A)** Low prognostic nutritional index (PNI) vs. high PNI (low PNI: ≤ 48.3, high PNI: > 48.3) and **(B)** low geriatric nutritional risk index (GNRI) vs. high GNRI (low GNRI: ≤ 97.04, high GNRI: > 97.04). The Kaplan–Meier method was used to calculate the survival rate, and the log-rank test was used to compare survival distributions between the groups.

**Figure 4:**
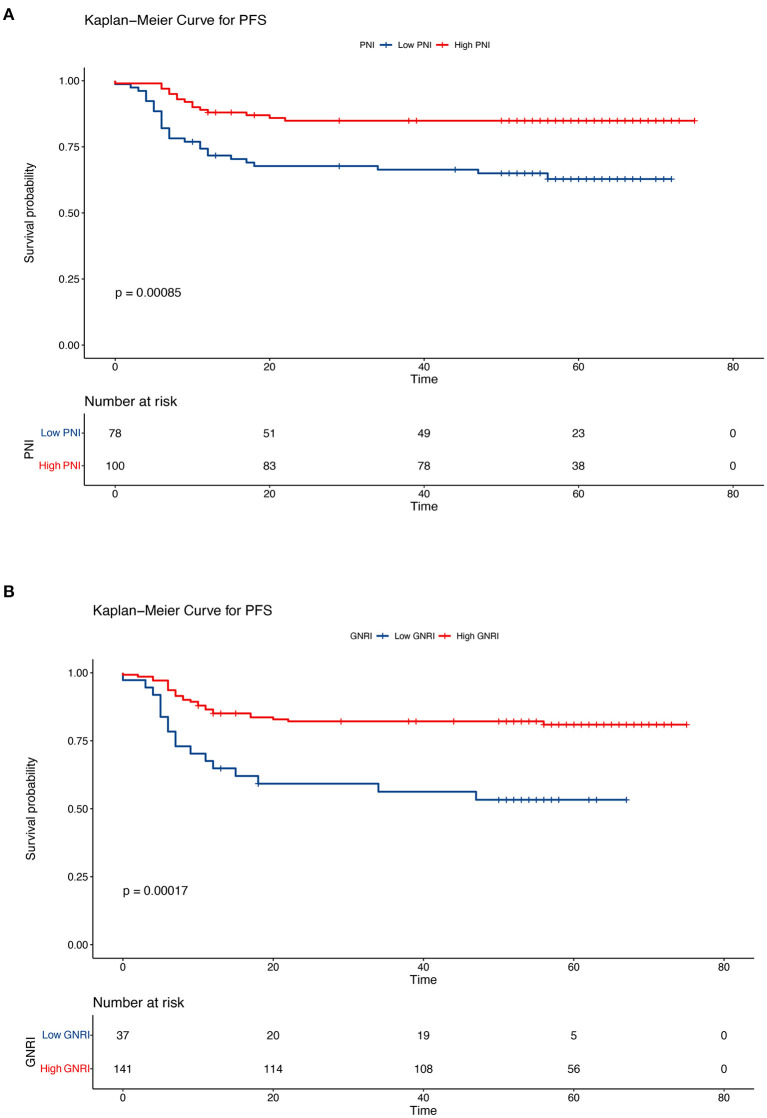
Kaplan–Meier curves of progression-free survival according to the nutritional indicators. **(A)** Low prognostic nutritional index (PNI) vs. high PNI (low PNI: ≤ 48.3, high PNI: > 48.3) and **(B)** low geriatric nutritional risk index (GNRI) vs. high GNRI (low GNRI: ≤ 97.04, high GNRI: > 97.04). The Kaplan–Meier method was used to calculate the survival rate, and the log-rank test was used to compare survival distributions between the groups.

In this retrospective cohort study, the 5-year OS rate of the entire population was 75.7%. The effect of nutritional status, as determined using the PNI and GNRI, on the prognosis of patients with cervical cancer was evaluated. Kaplan–Meier analysis showed that patients with a low PNI had shorter OS and PFS (low PNI vs. high PNI, 5-year OS, 64.1% vs. 84.9%, *P* < 0.001; 5-year PFS, 62.8% vs. 84.9%, *P* < 0.001) (Figures 3A, 4A). Similar results were obtained for the relationship between a low GNRI and the survival time of patients with cervical cancer (5-year OS, 48.5 vs. 82.2%, *P* < 0.001; 5-year PFS, 53.3 vs. 80.9%, *P* < 0.001) (Figures 3B, 4B). Survival analysis stratified by chemoradiotherapy (CRT) showed that patients with low PNI and GNRI values had shorter OS (low PNI vs. high PNI, *P* < 0.01; low GNRI vs. high GNRI, *P* < 0.001) and PFS (low PNI vs. high PNI, *P* < 0.001; low GNRI vs. high GNRI, *P* < 0.001) (Supplementary Figures 1, 2). Patients with a low GNRI had shorter OS (*P* < 0.05) and PFS (*P* < 0.05) than patients with a high GNRI in the survival analysis stratified by RT alone. There was no significant association between low PNI and OS/PFS in the survival analysis stratified by RT alone (Supplementary Figures 3, 4).

### 3.3. Prognostic value of NLR, MLR, and PLR

**Figure 5:**
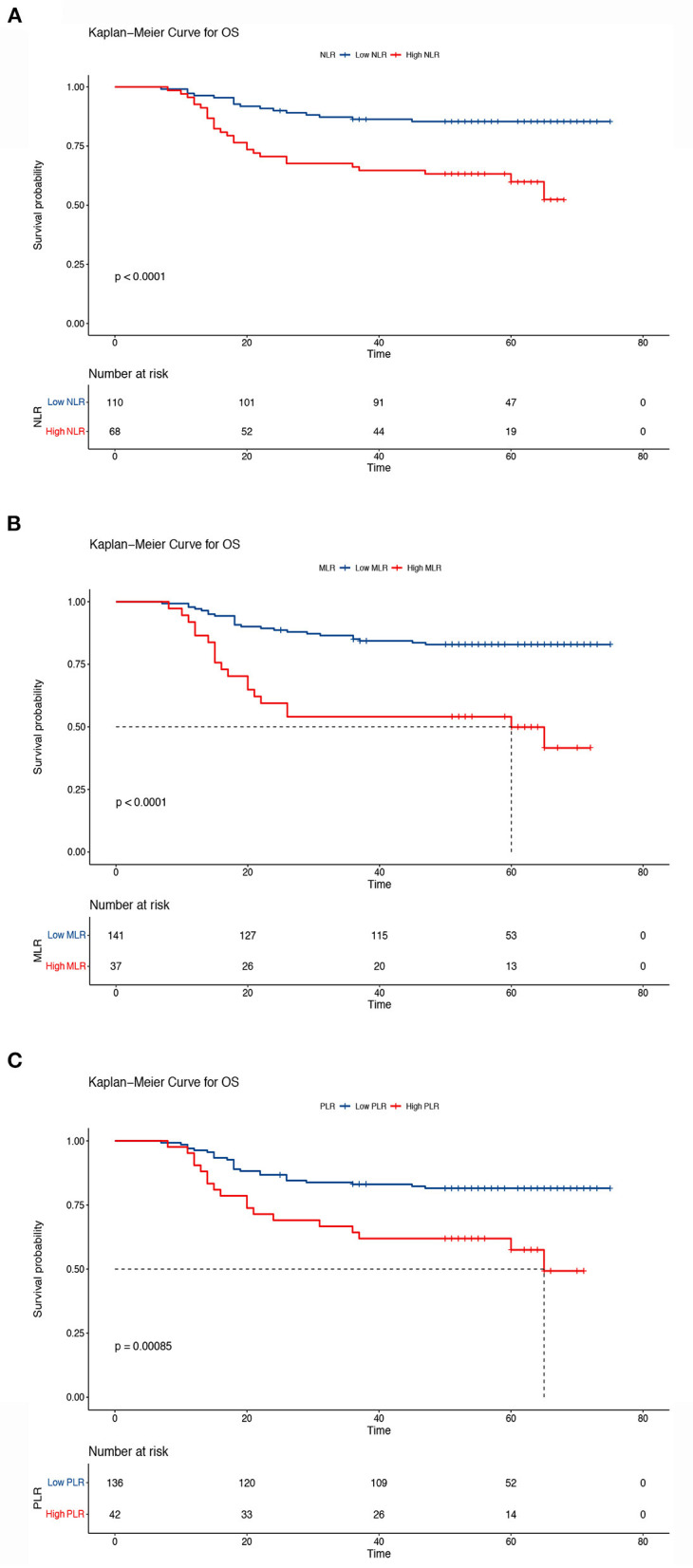
Kaplan–Meier curves of overall survival according to the inflammatory indicators. **(A)** Low neutrocyte/lymphocyte ratio (NLR) vs. high NLR (low NLR: ≤ 2.8, high NLR: > 2.8), **(B)** low monocyte/lymphocyte ratio (MLR) vs. high MLR (low MLR: ≤ 0.41, high MLR: > 0.41), and **(C)** low platelet lymphocyte ratio (PLR) vs. high PLR (low PLR: ≤ 186.67, high PLR: > 186.67). The Kaplan–Meier method was used to calculate the survival rate, and the log-rank test was used to compare survival distributions between the groups.

**Figure 6:**
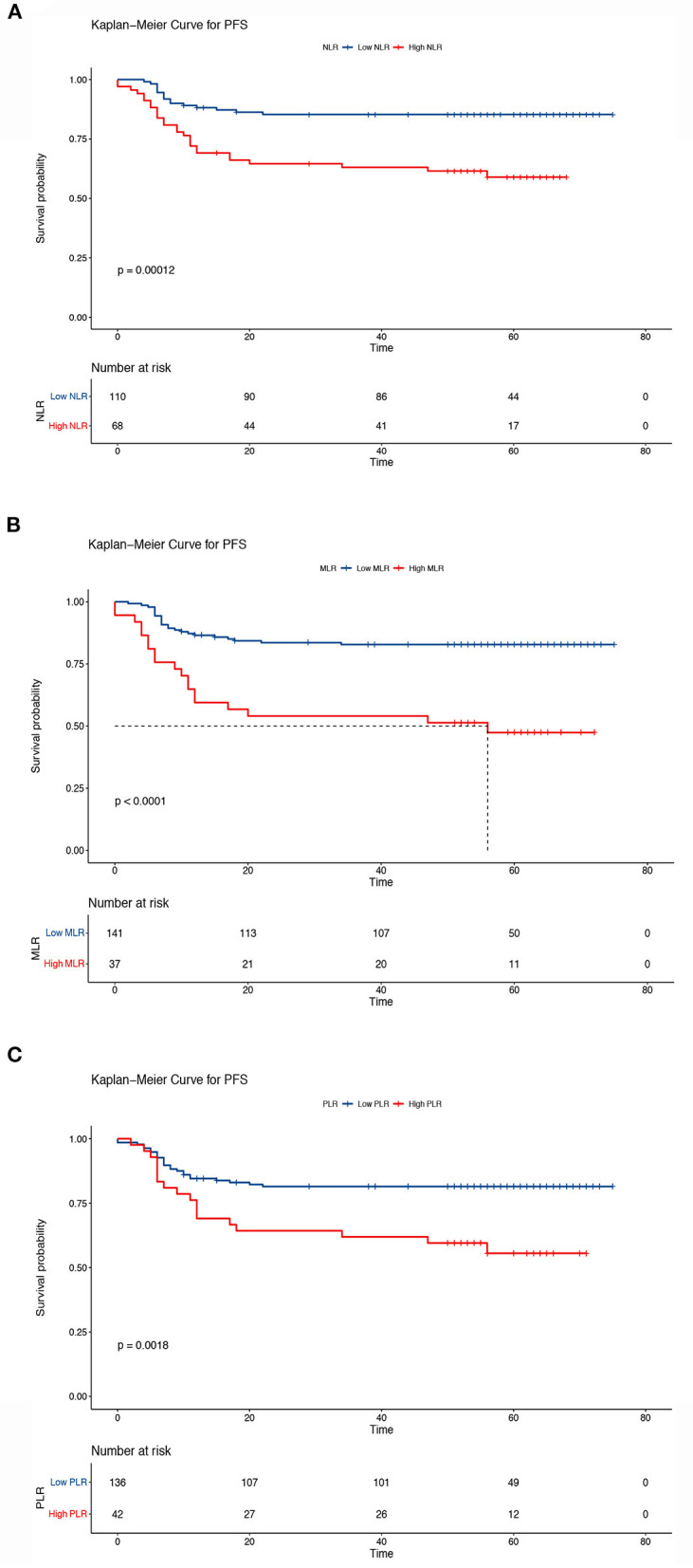
Kaplan–Meier curves of progression-free survival according to the inflammatory indicators. **(A)** Low neutrocyte/lymphocyte ratio (NLR) vs. high NLR (low NLR: ≤ 2.8, high NLR: > 2.8), **(B)** low monocyte/lymphocyte ratio (MLR) vs. high MLR (low MLR: ≤ 0.41, high MLR: > 0.41), and **(C)** low platelet/lymphocyte ratio (PLR) vs. high PLR (low PLR: ≤ 186.67, high PLR: > 186.67). The Kaplan–Meier method was used to calculate the survival rate, and the log-rank test was used to compare survival distributions between the groups.

The Kaplan–Meier results indicated that survival time differed depending on the NLR, MLR, and PLR. Patients with low NLR, MLR, and PLR had higher OS than patients in the other groups (5-year OS, low NLR vs. high NLR, 85.4 vs. 59.9%, *P* < 0.001; low MLR vs. high MLR, 82.9 vs. 49.9%, *P* < 0.001; low PLR vs. high PLR: 81.5 vs. 57.5%, *P* < 0.001) (Figure 5). We also analyzed the prognostic relationship between the systemic inflammatory indexes and PFS. Similar results were obtained (5-year PFS: low NLR vs. high NLR, 85.3 vs. 59.0%, *P* < 0.001; low MLR vs. high MLR, 82.8% vs. 47.4%, *P* < 0.001; low PLR vs. high PLR, 81.5 vs. 5.6%, *P* < 0.001) (Figure 6). A significant association between low NLR/MLP/PLR and higher OS/FPS was also found in the survival analysis stratified by CRT (OS, *P* < 0.001; PFS *P* < 0.001) (Supplementary Figures 1, 2). In the survival analysis stratified by RT alone, there was no significant association between low NLR/MLP/PLR and OS/PFS (Supplementary Figures 3, 4).

### 3.4. Univariate and multivariate analyses for patients with cervical cancer

Univariate and multivariate analyses of the baseline characteristics of OS and PFS are shown in Tables 2, 3. In univariate analysis, tumor size, histological type, stage, number of metastatic lymph nodes, PNI, GNRI, NLR, PLR, and MLR were significantly associated with poor OS and PFS. Other factors, including age, type of RT, SCC antigen levels, and body weight, had no effect on cervical cancer prognosis. In univariate Cox regression analysis, the number of metastatic lymph nodes, tumor size, histological type, stage, GNRI, NLR, and MLR were the most significant predictors of OS and PFS, with hazards ratios (HR) higher than 3 or < 0.33.

**Table 2:** Univariate and multivariate analysis for overall survival.

**Variables**	**Univariate analysis**		**Multivariate analysis**	
	**HR (95%CI)**	***P*** **-value**	**HR (95%CI)**	***P*** **-value**
**Age**				
≤ 55 vs. >55	0.65 (0.35–1.23)	0.185	0.71 (0.37–1.37)	0.309
**No. of metastatic lymph nodes**				
≤ 2 vs. >2	3.11 (1.66–5.83)	< 0.001	2.04 (1.04–3.98)	0.036
**Size of tumor**				
≤ 4 cm vs. >4 cm	3.34 (1.68–6.63)	0.001	2.34 (1.15–4.74)	0.019
**Type of radiotherapy**				
IMRT vs. RT	1.21 (0.66–2.21)	0.54	–	–
**Chemoradiotherapy**				
Yes vs. NO	0.51 (0.16–1.65)	0.26		
**Pathology**				
squamous cell carcinoma vs. adenocarcinoma	3.7 (1.82–7.52)	< 0.001	3.33 (1.59–7.00)	0.001
**FIGO Stage**				
II vs. III	3.25 (1.67–6.33)	0.001	2.33 (1.17–4.64)	0.016
**SCC**				
≤ 1.5 vs. > 1.5	1.24 (0.61–2.52)	0.547	–	–
**PNI**				
≤ 48.3 vs. > 48.3	0.35 (0.19–0.65)	0.001	0.47 (0.25~0.88)	0.019
**GNRI**				
≤ 97.04 vs. > 97.04	0.31 (0.17–0.57)	< 0.001	0.35 (0.18–0.68)	0.002
**NLR**				
≤ 2.8 vs. > 2.8	3.26 (1.75–6.06)	< 0.001	2.60 (1.36–4.97)	0.004
**MLR**				
≤ 0.41 vs. > 0.41	3.86 (2.11–7.05)	< 0.001	3.21 (1.66–6.23)	< 0.001
**PLR**				
≤ 186.67 vs. > 186.67	2.69 (1.47–4.93)	0.001	2.12 (1.09–4.13)	0.028

IMRT, Intensity-modulated radiotherapy; SCC, Squamous cell carcinoma; PNI, Prognostic nutritional index; GNRI, Geriatric nutritional risk index; NLR, Neutrophil/lymphocyte ratio; MLR, Monocyte/lymphocyte ratio; PLR, Platelet/lymphocyte ratio.

**Table 3:** Univariate and multivariate analysis for progression-free survival.

**Variables**	**Univariate analysis**		**Multivariate analysis**	
	**HR (95%CI)**	***P*** **-value**	**HR (95%CI)**	***P*** **-value**
**Age**				
≤ 55 vs. >55	0.66 (0.35–1.24)	0.201	–	–
**No. of metastatic lymph nodes**				
≤ 2 vs. >2	3.03 (1.62–5.67)	0.001	2.12 (1.10–4.09)	0.024
**Size of tumor**				
≤ 4 cm vs. >4 cm	3.28 (1.65–6.51)	0.001	2.31 (1.14–4.69)	0.020
**Type of radiotherapy**				
IMRT vs. RT	1.17 (0.64–2.14)	0.610	–	–
**Chemoradiotherapy**				
Yes vs. NO	0.46 (0.14–1.47)	0.20		
**Pathology**				
squamous cell carcinoma vs. adenocarcinoma	4.36 (2.14–8.88)	< 0.001	3.62 (1.75–7.49)	< 0.001
**FIGO Stage**				
II vs. III	3.28 (1.68–6.39)	< 0.001	2.34 (1.18–4.64)	0.015
**SCC antigen**				
≤ 1.5 vs. > 1.5	1.19 (0.59–2.41)	0.631	–	–
**PNI**				
≤ 48.3 vs. > 48.3	0.36 (0.19–0.67)	0.001	0.47 (0.28–0.87)	0.017
**GNRI**				
≤ 97.04 vs. > 97.04	0.33 (0.18–0.6)	< 0.001	0.34 (0.17–0.65)	0.001
**NLR**				
≤ 2.8 vs. > 2.8	3.15 (1.69–5.84)	< 0.001	2.66 (1.42–4.97)	0.002
**MLR**				
≤ 0.41 vs. > 0.41	3.73 (2.04–6.81)	< 0.001	3.36 (1.76–6.41)	< 0.001
**PLR**				
≤ 186.67 vs. > 186.67	2.55 (1.39–4.67)	0.002	2.05 (1.10–3.80)	0.023

IMRT, Intensity-modulated radiotherapy; SCC, Squamous cell carcinoma; PNI, Prognostic nutritional index; GNRI, Geriatric nutritional risk index; NLR, Neutrophil/lymphocyte ratio; MLR, Monocyte/lymphocyte ratio; PLR, Platelet/lymphocyte ratio.

In the multivariate Cox regression analysis, histological type remained the most significant predictor of OS (HR = 3.33; 95% confidence interval [CI], 1.59–7.00; *P* = 0.001) The multivariate analysis identified that PNI (HR = 0.47; 95% CI, 0.25–0.88; *P* < 0.01), GNRI (HR = 0.35; 95% CI, 0.18–0.68; *P* = 0.002), NLR (HR = 2.60; 95% CI, 1.36–4.97; *P* = 0.004), PLR (HR = 2.12; 95% CI, 1.09–4.13; *P* = 0.028), and MLR (HR = 3.21; 95% CI, 1.66–6.23, *P* < 0.001) were also significantly associated with OS. When the follow-up period was changed to PFS, PNI (HR = 0.47; 95%CI, 0.28–0.87; *P* = 0.017), GNRI (HR = 0.34; 95%CI, 0.17–0.65; *P* = 0.001), NLR (HR = 2.66; 95%CI, 1.42–4.97; *P* = 0.002), PLR (HR = 2.05; 95%CI, 1.10–3.80; *P* = 0.023), and MLR (HR = 3.36; 95%CI, 1.76–6.41; *P* < 0.001) were prognostic indicators for PFS, according to the multivariate analyses. In univariate and multivariate Cox regression analyses stratified by CRT, the GNRI, NLR, PLR, and MLR were also prognostic indicators for OS and PFS (Supplementary Tables 1, 2).

### 3.5. Prognostic nomograms of PNI, GNRI, and systemic inflammatory indexes

**Figure 7:**
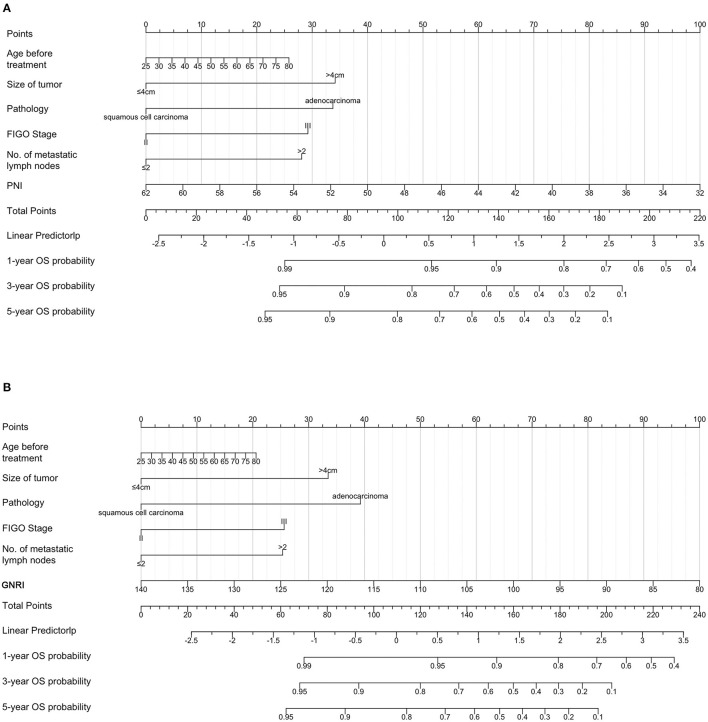
Prognostic nomograms for overall survival prediction according to the prognostic nutritional index (PNI) **(A)** and geriatric nutritional risk index (GNRI) **(B)**. Points were assigned for age before treatment, and for tumor size, histological type, stage, number of metastatic lymph nodes, and nutritional indicators. The score of each predictor was determined by drawing a vertical line from the value to the score scale. The total score was summed up by the scores of these predictors, which correspond to overall survival rate.

**Figure 8:**
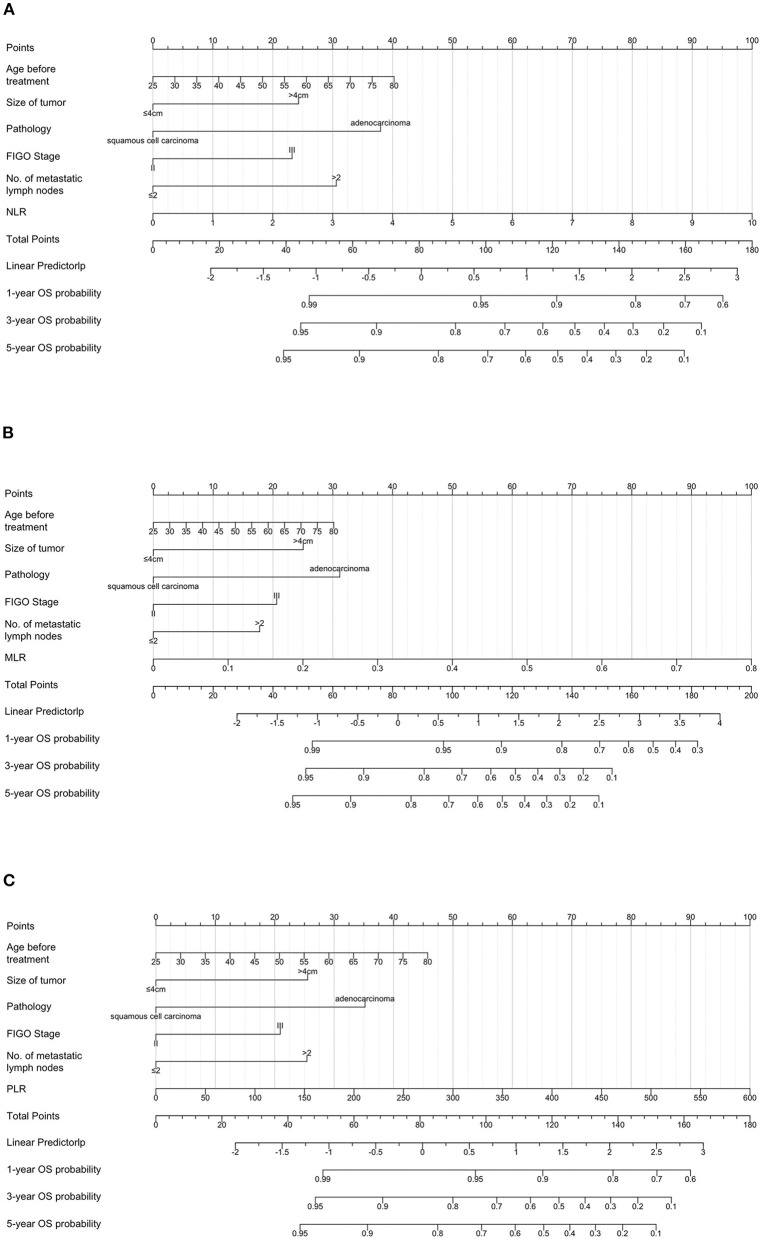
Prognostic nomograms for overall survival prediction according to the neutrophil/lymphocyte ratio (NLR) **(A)**, monocyte/lymphocyte ratio (MLR) **(B)**, and platelet/lymphocyte ratio (PLR) **(C)**. Points were assigned for age before treatment, and for tumor size, histological type, stage, number of metastatic lymph nodes, and inflammatory indicators. The score of each predictor was determined by drawing a vertical line from the value to the score scale. The total score was summed up by the scores of these predictors, which correspond to overall survival rate.

To predict the 3-year and 5-year OS of patients with cervical cancer, nomograms were constructed. Based on the results of the multivariate Cox analysis, the prognostic nomogram included tumor size, histological type, stage, number of metastatic lymph nodes, and PNI/GNRI/systemic inflammatory indexes (Figures 7, 8).

## 4. Discussion

For patients with stage IIB–III cervical cancer, RT and a combination of chemotherapy and RT are the suggested treatment options. The present study demonstrated that a low PNI, low GNRI, high NLR, high MLR, and high PLR were negative prognostic factors for survival in patients with stage IIB–III disease treated with RT.

Similar to other types of cancers, there is a high prevalence of malnutrition among patients with cervical cancer (35). The incidence of malnutrition was reported as high as 38.79% in patients undergoing cervical cancer surgery before treatment (36). Additionally, a higher stage grade indicates a higher incidence of malnutrition in cervical cancer (37). Poor nutritional status at baseline is also associated with poor quality of life and chemotherapy interruption in patients with cervical cancer (38). In clinical practice, the GNRI and PNI are easily obtained, objective, simple, efficient, and applicable tools to reflect nutritional status compared with other methods, such as patient-generated subjective global assessment and mini nutritional assessment. Our results also showed that poor status, as determined by the PNI and GNRI, was associated with shorter OS and PFS. Robust and consistent evidence has shown that cancer-related malnutrition plays a negative role in the prognosis of patients (39–42). Studies have shown that the prevalence of malnutrition in patients with cancer is as high as 80.4% before treatment, and that nutritional status worsens with the progression of anticancer therapies (43, 44). Due to clinically distinct causes, such as dysphagia, stomatitis, bowel obstruction caused by the tumor, and gastrointestinal disorders induced by anticancer therapies, the nutrient intake of patients with cancer is generally reduced (45). In addition, altered metabolism-induced by excess catabolism, anabolic resistance, inflammation caused by tumors, and cancer therapy significantly affect nutritional status (46). These factors lead to weight loss and skeletal muscle depletion in patients with cancer, which are independent risk factors for an unfavorable prognosis. Studies have demonstrated that unintentional weight loss is associated with poor post-operative survival and increased mortality risk in patients with cancer (47–49). The patients with locally advanced cervical cancer receiving primary chemoradiation who had unintentional weight loss ≥10% also had a higher risk of death (HR = 2.37) (50). Decreased skeletal muscle mass, widely known as sarcopenia, has also been closely associated with a poor quality of life and short life expectancy (51). Additionally, the common side effects of cytotoxic chemotherapy and RT directly affect the nutritional status of patients, and a poor nutritional status may aggravate these side effects (52). Moreover, the decreased clearance of antitumor drugs in the tissues of patients with malnutrition with a higher drug concentration in the tissue may also lead to a higher rate of treatment toxicity (53). The deterioration of nutritional status can lead to decreased treatment completion (54). Furthermore, loss of body weight with a specific loss of skeletal muscle combined with systemic inflammation caused by tumors results in cancer cachexia (55). Patients with cancer with cachexia have an impaired quality of life, high mortality, and increased treatment costs (46) and currently no effective medical intervention has been confirmed to completely reverse cachexia (56).

An increasing number of studies have shown that cancer-associated systemic inflammatory markers, such as NLR, PLR, and MLR, can be useful in predicting tumor progression. These markers are easily obtained, noninvasive, and inexpensive. Recently, three studies have demonstrated that systemic inflammatory markers are novel independent prognostic factors for predicting post-operative survival in patients with cervical cancer. High NLR, PLR, and MLR are closely related to poor prognosis (29–31). Similarly, our results showed that patients with stage IIB–III cervical cancer who underwent RT with high NLR, PLR, and MLR had shorter OS times. The close relationship between NLR/MLR and tumor prognosis involves tumor-induced inflammation and immune function changes. The systemic inflammatory response in patients with tumors is often accompanied by an increase in circulating neutrophil counts (57). Recent studies have found that neutrophils not only exert an anti-tumor effect, but also promote tumor progression (58). Most studies suggest that elevated neutrophil levels lead to tumor progression. The possible mechanisms by which neutrophils promote tumor progression include changes in the microenvironment shaped by cancer cells and release of some growth factors, such as epidermal growth factor and hepatocyte growth factor (59). Monocytes also have diverse functions in different types and stages of the tumor (60). The direct tumoricidal functions of monocytes result from cytokine-mediated induction of cell death and phagocytosis and effects on the components of the tumor microenvironment (61). Interestingly, our study also suggests that low PLR is associated with cervical cancer prognosis. This result was inconsistent with Li's finding that PLR was not a significant independent prognostic factor in patients with stage IIB cervical cancer (33). Another study also found that PLR was not associated with OS in gynecological cancer (62). The inconsistent results may be due to the different stages of patients included in the different studies, which could affect the prognosis of cervical cancer. As an essential component of the blood, platelets play an important role in the inflammatory response in patients with cancer with chronic inflammation (63). Angiogenesis is facilitated by the release of pro-angiogenic proteins, such as vascular epidermal growth factor and transforming growth factor, in the tumor microenvironment. The cytokines released by platelets can induce cancer-related inflammation and promote tumor growth and invasion (57).

Many studies have demonstrated that concurrent chemoradiotherapy provides therapeutic benefits over RT alone (64). To explore the prognostic value of the PNI, GNRI, and systemic inflammatory indexes in patients who underwent CRT and RT alone, we performed survival analyses, univariate and multivariate analyses stratified by RT or CRT. The results showed that low GNRI, high NLR, high MLR, and high PLR predicted worse prognosis in patients treated with CRT. However, similar results were not observed in the patients who received RT alone. These inconsistent results may be explained by the small number of patients who underwent RT alone. Although there was an association between low PNI and poor OS/PFS in the multivariate cox analysis for all the patients, this association was not statistically significant in the multivariate analyses stratified by CRT. The possible reason is that patients who can only receive radiotherapy alone have poorer nutritional status than those who can receive concurrent chemotherapy.

Our study has several limitations. First, this was a retrospective study, and all data were collected from a single center. Second, the inflammatory state induced by infection before treatment may have an impact on the outcome. Third, we were not able to evaluate all covariates that might have affected prognosis, even though we included all likely covariates. Moreover, the sample size in this study was small. Additional prospective cohort studies are needed to determine the effects of GNRI, PNI, and systemic inflammatory indexes in patients with stage IIB–III cervical cancer.

## 5. Conclusions

Pretreatment GNRI, PNI, and systemic inflammatory indexes might be novel prognostic predictors for patients with stage II–III cervical cancer treated with RT. Low PNI, low GNRI, high NLR, high MLR, and high PLR predicted a worse prognosis. These markers can be incorporated into pretreatment evaluations and act as factors for decision-making in patients with cervical cancer receiving radiotherapy.

## Data availability statement

The raw data supporting the conclusions of this article will be made available by the authors, without undue reservation.

## Ethics statement

The studies involving human participants were reviewed and approved by Ethics Committee of Hubei Cancer Hospital of Huazhong University of Science and Technology. The Ethics Committee waived the requirement of written informed consent for participation.

## Author contributions

XJ: conceptualization, methodology, software, investigation, and writing-original draft. H-BW: data collection and writing-review and editing. X-TX: methodology, software, and investigation. M-XT: resources, data curation, and investigation. C-CD, JT, and YQ: writing-review and editing. All authors revised and approved the final manuscript.

## References

[1] Bray F Ferlay J Soerjomataram I Siegel RL Torre LA Jemal A. Global cancer statistics 2018: globocan estimates of incidence and mortality worldwide for 36 cancers in 185 countries. CA Cancer J Clin. (2018) 68:394–424. 10.3322/caac.21492 30207593

[2] Sung H Ferlay J Siegel RL Laversanne M Soerjomataram I Jemal A. Global cancer statistics 2020: globocan estimates of incidence and mortality worldwide for 36 cancers in 185 countries. CA Cancer J Clin. (2021) 71:209–49. 10.3322/caac.21660 33538338

[3] Vizcaino AP Moreno V Bosch FX Munoz N Barros-Dios XM Borras J. International trends in incidence of cervical cancer: Ii. Squamous-Cell Carcinoma Int J Cancer. (2000) 86:429–35. 10.1002/(SICI)1097-0215(20000501)86:3<429::AID-IJC20>3.0.CO;2-D 10760834

[4] Shrivastava S Mahantshetty U Engineer R Tongaonkar H Kulkarni J Dinshaw K. Treatment and outcome in cancer cervix patients treated between 1979 and 1994: a single institutional experience. J Cancer Res Ther. (2013) 9:672–9. 10.4103/0973-1482.126480 24518716

[5] Manders DB Moron A McIntire D Miller DS Richardson DL Kehoe SM. Locally advanced cervical cancer: outcomes with variable adherence to treatment. Am J Clin Oncol. (2018) 41:447–51. 10.1097/COC.0000000000000300 27258678

[6] Ditto A Martinelli F Lo Vullo S Reato C Solima E Carcangiu M. The role of lymphadenectomy in cervical cancer patients: the significance of the number and the status of lymph nodes removed in 526 cases treated in a single institution. Ann Surg Oncol. (2013) 20:3948–54. 10.1245/s10434-013-3067-6 23812772

[7] Huang Y Zou D Guo M He M He H Li X. Hpv and radiosensitivity of cervical cancer: a narrative review. Ann Transl Med. (2022) 10:1405. 10.21037/atm-22-5930 36660629 PMC9843372

[8] Bhatla N Aoki D Sharma DN Sankaranarayanan R. Cancer of the Cervix Uteri. Int J Gynaecol Obstet. (2018) 143 Suppl 2:22–36. 10.1002/ijgo.12611 30306584

[9] Cho O Chun M. Management for locally advanced cervical cancer: new trends and controversial issues. Radiat Oncol J. (2018) 36:254–64. 10.3857/roj.2018.00500 30630264 PMC6361251

[10] Williams NL Werner TL Jarboe EA Gaffney DK. Adenocarcinoma of the cervix: should we treat it differently? Curr Oncol Rep. (2015) 17:17. 10.1007/s11912-015-0440-6 25708801

[11] Hu K Wang W Liu X Meng Q Zhang F. Comparison of treatment outcomes between squamous cell carcinoma and adenocarcinoma of cervix after definitive radiotherapy or concurrent chemoradiotherapy. Radiat Oncol. (2018) 13:249. 10.1186/s13014-018-1197-5 30558636 PMC6296025

[12] Cong M Zhu W Wang C Fu Z Song C Dai Z. Nutritional status and survival of 8247 cancer patients with or without diabetes mellitus-results from a prospective cohort study. Cancer Med. (2020) 9:7428–39. 10.1002/cam4.3397 32813914 PMC7571830

[13] Fujiya K Kawamura T Omae K Makuuchi R Irino T Tokunaga M. Impact of malnutrition after gastrectomy for gastric cancer on long-term survival. Ann Surg Oncol. (2018) 25:974–83. 10.1245/s10434-018-6342-8 29388124

[14] Santos I Mendes L Mansinho H Santos CA. Nutritional status and functional status of the pancreatic cancer patients and the impact of adjacent symptoms. Clin Nutr. (2021) 40:5486–93. 10.1016/j.clnu.2021.09.019 34656030

[15] Kubrak C Martin L Gramlich L Scrimger R Jha N Debenham B. Prevalence and prognostic significance of malnutrition in patients with cancers of the head and neck. Clin Nutr. (2020) 39:901–9. 10.1016/j.clnu.2019.03.030 31000341

[16] Zhang X Tang M Zhang Q Zhang KP Guo ZQ Xu HX. The glim criteria as an effective tool for nutrition assessment and survival prediction in older adult cancer patients. Clin Nutr. (2021) 40:1224–32. 10.1016/j.clnu.2020.08.004 32826109

[17] Xu XT He DL Tian MX Wu HJ Jin X. Prognostic value of sarcopenia in patients with diffuse large B-Cell lymphoma treated with R-chop: a systematic review and meta-analysis. Front Nutr. (2022) 9:816883. 10.3389/fnut.2022.816883 35284466 PMC8914205

[18] Kim SI Kim SJ Kim SJ Cho DS. Prognostic nutritional index and prognosis in renal cell carcinoma: a systematic review and meta-analysis. Urol Oncol. (2021) 39:623–30. 10.1016/j.urolonc.2021.05.028 34253447

[19] Bullock AF Greenley SL McKenzie GAG Paton LW Johnson MJ. Relationship between markers of malnutrition and clinical outcomes in older adults with cancer: systematic review, narrative synthesis and meta-analysis. Eur J Clin Nutr. (2020) 74:1519–35. 10.1038/s41430-020-0629-0 32366995 PMC7606134

[20] Wang D Hu X Xiao L Long G Yao L Wang Z. Prognostic nutritional index and systemic immune-inflammation index predict the prognosis of patients with Hcc. J Gastrointest Surg. (2021) 25:421–7. 10.1007/s11605-019-04492-7 32026332 PMC7904713

[21] Buzby GP Mullen JL Matthews DC Hobbs CL Rosato EF. Prognostic nutritional index in gastrointestinal surgery. Am J Surg. (1980) 139:160–7. 10.1016/0002-9610(80)90246-9 7350839

[22] Yan L Nakamura T Casadei-Gardini A Bruixola G Huang YL Hu ZD. Long-term and short-term prognostic value of the prognostic nutritional index in cancer: a narrative review. Ann Transl Med. (2021) 9:1630. 10.21037/atm-21-4528 34926674 PMC8640913

[23] Park S Ahn HJ Yang M Kim JA Kim JK Park SJ. The prognostic nutritional index and post-operative complications after curative lung cancer resection: a retrospective cohort study. J Thorac Cardiovasc Surg. (2020) 160:276–85 e1. 10.1016/j.jtcvs.2019.10.105 31859072

[24] Karayama M Inoue Y Yasui H Hozumi H Suzuki Y Furuhashi K. Association of the geriatric nutritional risk index with the survival of patients with non-small-cell lung cancer after platinum-based chemotherapy. BMC Pulm Med. (2021) 21:409. 10.1186/s12890-021-01782-2 34895201 PMC8665565

[25] Kumarasamy C Sabarimurugan S Madurantakam RM Lakhotiya K Samiappan S Baxi S. Prognostic significance of blood inflammatory biomarkers Nlr, Plr, and Lmr in cancer-a protocol for systematic review and meta-analysis. Medicine. (2019) 98:e14834. 10.1097/MD.0000000000014834 31192906 PMC6587598

[26] Diem S Schmid S Krapf M Flatz L Born D Jochum W. Neutrophil-to-lymphocyte ratio (Nlr) and platelet-to-lymphocyte ratio (Plr) as prognostic markers in patients with non-small cell lung cancer (Nsclc) treated with nivolumab. Lung Cancer. (2017) 111:176–81. 10.1016/j.lungcan.2017.07.024 28838390

[27] Kang Y Zhu X Lin Z Zeng M Shi P Cao Y. Compare the diagnostic and prognostic value of mlr, Nlr and Plr in Crc patients. Clin Lab. (2021) 67:9. 10.7754/Clin.Lab.2021.201130 34542964

[28] Cho SH Hwang JE Bae WK Chung IJ. The prognostic role of Pd-L1 expression according to Msi status in stage Iii colon cancer after curative resection. J Clin Oncol. (2019) 37:555. 10.1200/JCO.2019.37.4_suppl.555

[29] Huang H Liu Q Zhu L Zhang Y Lu X Wu Y. Prognostic value of preoperative systemic immune-inflammation index in patients with cervical cancer. Sci Rep. (2019) 9:3284. 10.1038/s41598-019-39150-0 30824727 PMC6397230

[30] Chao B Ju X Zhang L Xu X Zhao Y A. Novel prognostic marker systemic inflammation response index (Siri) for operable cervical cancer patients. Front Oncol. (2020) 10:766. 10.3389/fonc.2020.00766 32477958 PMC7237698

[31] Deng Q Long Q Liu Y Yang Z Du Y Chen X. Prognostic value of preoperative peripheral blood mean platelet volume/platelet count ratio (Mpv/Pc) in patients with resectable cervical cancer. BMC Cancer. (2021) 21:1282. 10.1186/s12885-021-09016-8 34844568 PMC8628453

[32] Jiang Y Gu H Zheng X Pan B Liu P Zheng M. Pretreatment C-reactive protein/albumin ratio is associated with poor survival in patients with 2018 Figo Stage Ib-Iia Hpv-positive cervical cancer. Pathol Oncol Res. (2021) 27:1609946. 10.3389/pore.2021.1609946 34992504 PMC8724028

[33] Li YX Chang JY He MY Wang HR Luo DQ Li FH. Neutrophil-to-lymphocyte ratio (Nlr) and monocyte-to-lymphocyte ratio (Mlr) predict clinical outcome in patients with stage Iib cervical cancer. J Oncol. (2021) 2021:2939162. 10.1155/2021/2939162 34539781 PMC8443385

[34] Haraga J Nakamura K Omichi C Nishida T Haruma T Kusumoto T. Pretreatment prognostic nutritional index is a significant predictor of prognosis in patients with cervical cancer treated with concurrent chemoradiotherapy. Mol Clin Oncol. (2016) 5:567–74. 10.3892/mco.2016.1028 27900086 PMC5103867

[35] Bossi P Delrio P Mascheroni A Zanetti M. The spectrum of malnutrition/cachexia/sarcopenia in oncology according to different cancer types and settings: a narrative review. Nutrients. (2021) 13:1980. 10.3390/nu13061980 34207529 PMC8226689

[36] Tian M Fu H Du J. Application value of Nrs2002 and Pg-Sga in nutritional assessment for patients with cervical cancer surgery. Am J Transl Res. (2021) 13:7186–92. 34306480 PMC8290634

[37] Flores-Cisneros L Cetina-Perez L Castillo-Martinez L Jimenez-Lima R Luvian-Morales J Fernandez-Loaiza M. Body composition and nutritional status according to clinical stage in patients with locally advanced cervical cancer. Eur J Clin Nutr. (2021) 75:852–5. 10.1038/s41430-020-00797-y 33149254

[38] Aredes MA Garcez MR Chaves GV. Influence of chemoradiotherapy on nutritional status, functional capacity, quality of life and toxicity of treatment for patients with cervical cancer. Nutr Diet. (2018) 75:263–70. 10.1111/1747-0080.12414 29464856

[39] Laviano A Di Lazzaro L Koverech A. Nutrition support and clinical outcome in advanced cancer patients. Proc Nutr Soc. (2018) 77:388–93. 10.1017/S0029665118000459 30001763

[40] Arends J Bachmann P Baracos V Barthelemy N Bertz H Bozzetti F. Espen guidelines on nutrition in cancer patients. Clin Nutr. (2017) 36:11–48. 10.1016/j.clnu.2016.07.015 27637832

[41] Aaldriks AA van der Geest LG Giltay EJ. le Cessie S, Portielje JE, Tanis BC, et al. Frailty and malnutrition predictive of mortality risk in older patients with advanced colorectal cancer receiving chemotherapy. J Geriatr Oncol. (2013) 4:218–26. 10.1016/j.jgo.2013.04.001 24070460

[42] Fukuda Y Yamamoto K Hirao M Nishikawa K Maeda S Haraguchi N. Prevalence of malnutrition among gastric cancer patients undergoing gastrectomy and optimal preoperative nutritional support for preventing surgical site infections. Ann Surg Oncol. (2015) 22:S778–85. 10.1245/s10434-015-4820-9 26286199

[43] Baracos VE. Cancer-associated malnutrition. Eur J Clin Nutr. (2018) 72:1255–9. 10.1038/s41430-018-0245-4 30185853

[44] Guo ZQ Yu JM Li W Fu ZM Lin Y Shi YY. Survey and analysis of the nutritional status in hospitalized patients with malignant gastric tumors and its influence on the quality of life. Support Care Cancer. (2020) 28:373–80. 10.1007/s00520-019-04803-3 31049672 PMC6882767

[45] Martin L Kubrak C. How much does reduced food intake contribute to cancer-associated weight loss? Curr Opin Support Palliat Care. (2018) 12:410–9. 10.1097/SPC.0000000000000379 30124527

[46] Baracos VE Martin L Korc M Guttridge DC Fearon KCH. Cancer-associated cachexia. Nat Rev Dis Primers. (2018) 4:17105. 10.1038/nrdp.2017.105 29345251

[47] Hue JJ Sugumar K Kyasaram RK Shanahan J Lyons J Ocuin LM. Weight loss as an untapped early detection marker in pancreatic and periampullary cancer. Ann Surg Oncol. (2021) 28:6283–92. 10.1245/s10434-021-09861-8 33835301

[48] Paixao EMS Gonzalez MC Nakano EY Ito MK Pizato N. Weight loss, phase angle, and survival in cancer patients undergoing radiotherapy: a prospective study with 10-year follow-up. Eur J Clin Nutr. (2021) 75:823–8. 10.1038/s41430-020-00799-w 33177697

[49] Hendifar AE Petzel MQB Zimmers TA Denlinger CS Matrisian LM Picozzi VJ. Pancreas cancer-associated weight loss. Oncologist. (2019) 24:691–701. 10.1634/theoncologist.2018-0266 30591550 PMC6516128

[50] Jou J Coulter E Roberts T Binder P Saenz C McHale M. Assessment of malnutrition by unintentional weight loss and its implications on oncologic outcomes in patient with locally advanced cervical cancer receiving primary chemoradiation. Gynecol Oncol. (2021) 160:721–8. 10.1016/j.ygyno.2020.12.009 33342621

[51] Muscaritoli M Anker SD Argiles J Aversa Z Bauer JM Biolo G. Consensus definition of sarcopenia, cachexia and pre-cachexia: joint document elaborated by special interest groups (Sig) “cachexia-anorexia in chronic wasting diseases” and “nutrition in geriatrics”. Clin Nutr. (2010) 29:154–9. 10.1016/j.clnu.2009.12.004 20060626

[52] Laura FC Lucely CP Tatiana GC Roberto JL Dulce GI Arturo PS. Handgrip strength, overhydration and nutritional status as a predictors of gastrointestinal toxicity in cervical cancer patients. A prospective study. Nutr Cancer. (2022) 74:2444–50. 10.1080/01635581.2021.2012209 35023398

[53] Prado CM Lima IS Baracos VE Bies RR McCargar LJ Reiman T. An exploratory study of body composition as a determinant of epirubicin pharmacokinetics and toxicity. Cancer Chemother Pharmacol. (2011) 67:93–101. 10.1007/s00280-010-1288-y 20204364

[54] Hamaker ME Oosterlaan F van Huis LH Thielen N Vondeling A van den Bos F. Nutritional status and interventions for patients with cancer—a systematic review. J Geriatr Oncol. (2021) 12:6–21. 10.1016/j.jgo.2020.06.020 32616384

[55] Fearon K Strasser F Anker SD Bosaeus I Bruera E Fainsinger RL. Definition and classification of cancer cachexia: an international consensus. Lancet Oncol. (2011) 12:489–95. 10.1016/S1470-2045(10)70218-7 21296615

[56] Jin X Xu XT Tian MX Dai Z. Omega-3 polyunsaterated fatty acids improve quality of life and survival, but not body weight in cancer cachexia: a systematic review and meta-analysis of controlled trials. Nutr Res. (2022) 107:165–78. 10.1016/j.nutres.2022.09.009 36283229

[57] Yamamoto T Kawada K Obama K. Inflammation-related biomarkers for the prediction of prognosis in colorectal cancer patients. Int J Mol Sci. (2021) 22:8002. 10.3390/ijms22158002 34360768 PMC8348168

[58] Kargl J Busch SE Yang GH Kim KH Hanke ML Metz HE. Neutrophils dominate the immune cell composition in non-small cell lung cancer. Nat Commun. (2017) 8:14381. 10.1038/ncomms14381 28146145 PMC5296654

[59] Xiong S Dong L Cheng L. Neutrophils in cancer carcinogenesis and metastasis. J Hematol Oncol. (2021) 14:173. 10.1186/s13045-021-01187-y 34674757 PMC8529570

[60] Olingy CE Dinh HQ Hedrick CC. Monocyte heterogeneity and functions in cancer. J Leukoc Biol. (2019) 106:309–22. 10.1002/JLB.4RI0818-311R 30776148 PMC6658332

[61] Robinson A Han CZ Glass CK Pollard JW. Monocyte regulation in homeostasis and malignancy. Trends Immunol. (2021) 42:104–19. 10.1016/j.it.2020.12.001 33446416 PMC7877795

[62] Abu-Shawer O Abu-Shawer M Hirmas N Alhouri A Massad A Alsibai B. Hematologic markers of distant metastases and poor prognosis in gynecological cancers. BMC Cancer. (2019) 19:141. 10.1186/s12885-019-5326-9 30755184 PMC6373103

[63] Wagner DD. New Links between inflammation and thrombosis. Arterioscler Thromb Vasc Biol. (2005) 25:1321–4. 10.1161/01.ATV.0000166521.90532.44 15831808

[64] Datta NR Stutz E Liu M Rogers S Klingbiel D Siebenhuner A. Concurrent chemoradiotherapy vs. radiotherapy alone in locally advanced cervix cancer: a systematic review and meta-analysis. Gynecol Oncol. (2017) 145:374–85. 10.1016/j.ygyno.2017.01.033 28188016

