# Supplementary material for: Prognostic values of the prognostic nutritional index, geriatric nutritional risk index, and systemic inflammatory indexes in patients with stage IIB–III cervical cancer receiving radiotherapy
Source: Front Nutr. 2023 Mar 2;10:1000326. doi: 10.3389/fnut.2023.1000326 (PMC10017984; doi:10.3389/fnut.2023.1000326)
Supplement: Supplementary file 1 [file Data_Sheet_1.docx]

Supplementary Material

# Supplementary Figures and Tables

## Supplementary Figures


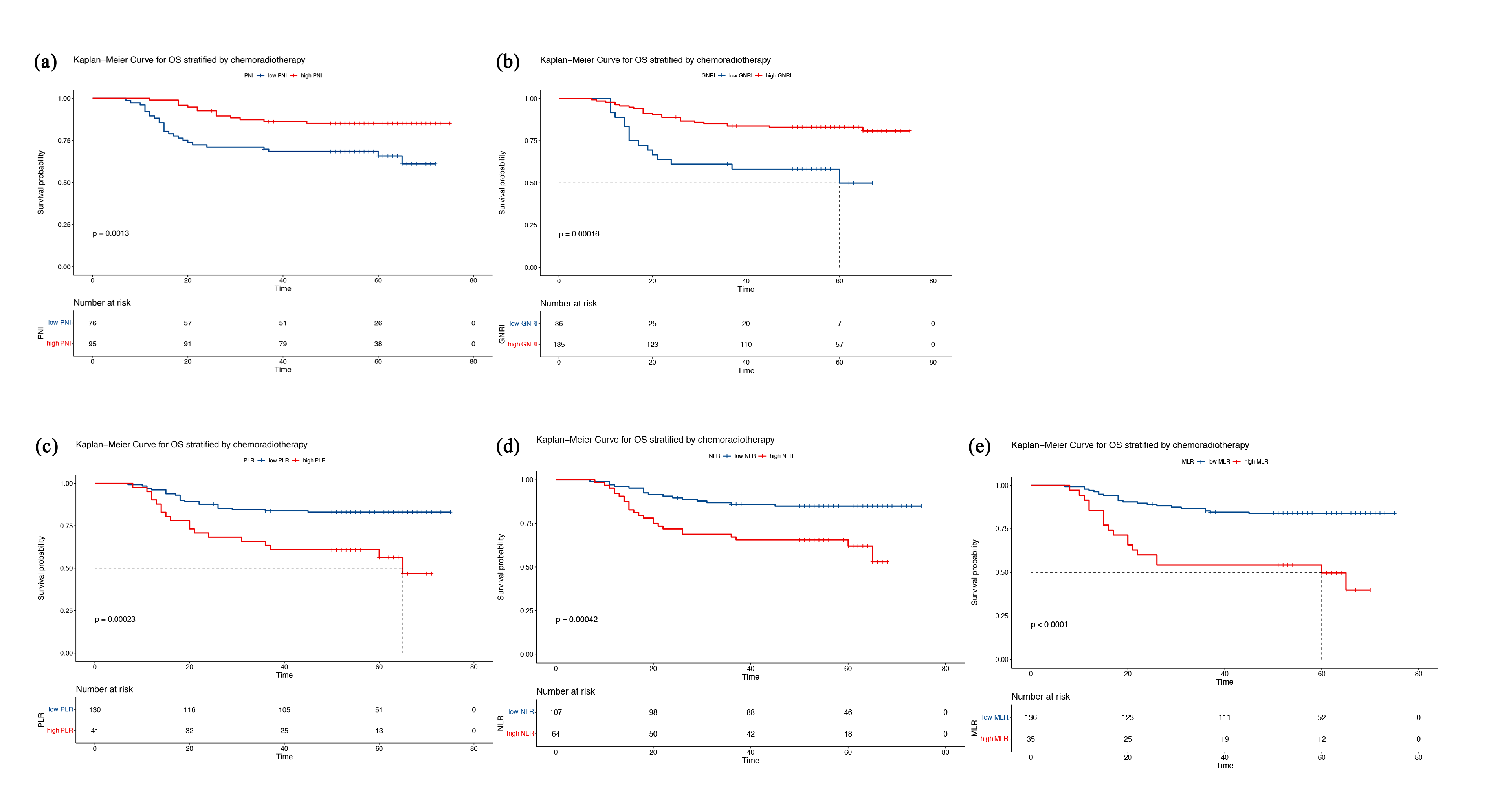


**Supplemental Figure 1.** Kaplan–Meier curves of overall survival stratified by chemoradiotherapy (CRT) according to nutritional and inflammatory indicators. (a) Low prognostic nutritional index (PNI) vs. high PNI (low PNI: ≤ 48.3, high PNI: > 48.3), (b) low geriatric nutritional risk index (GNRI) vs. high GNRI (low GNRI: ≤ 97.04, high GNRI: > 97.04), (c) low platelet/lymphocyte ratio (PLR) vs. high PLR (low PLR: ≤ 186.67, high PLR: > 186.67), (d) low neutrophil/lymphocyte ratio (NLR) vs. high NLR (low NLR: ≤ 2.8, high NLR: > 2.8), and (e) low monocyte/lymphocyte ratio (MLR) vs. high MLR (low MLR: ≤ 0.41, high MLR: > 0.41). The Kaplan–Meier method was used to calculate the survival rate, and the log-rank test was used to compare the survival distributions between the groups.


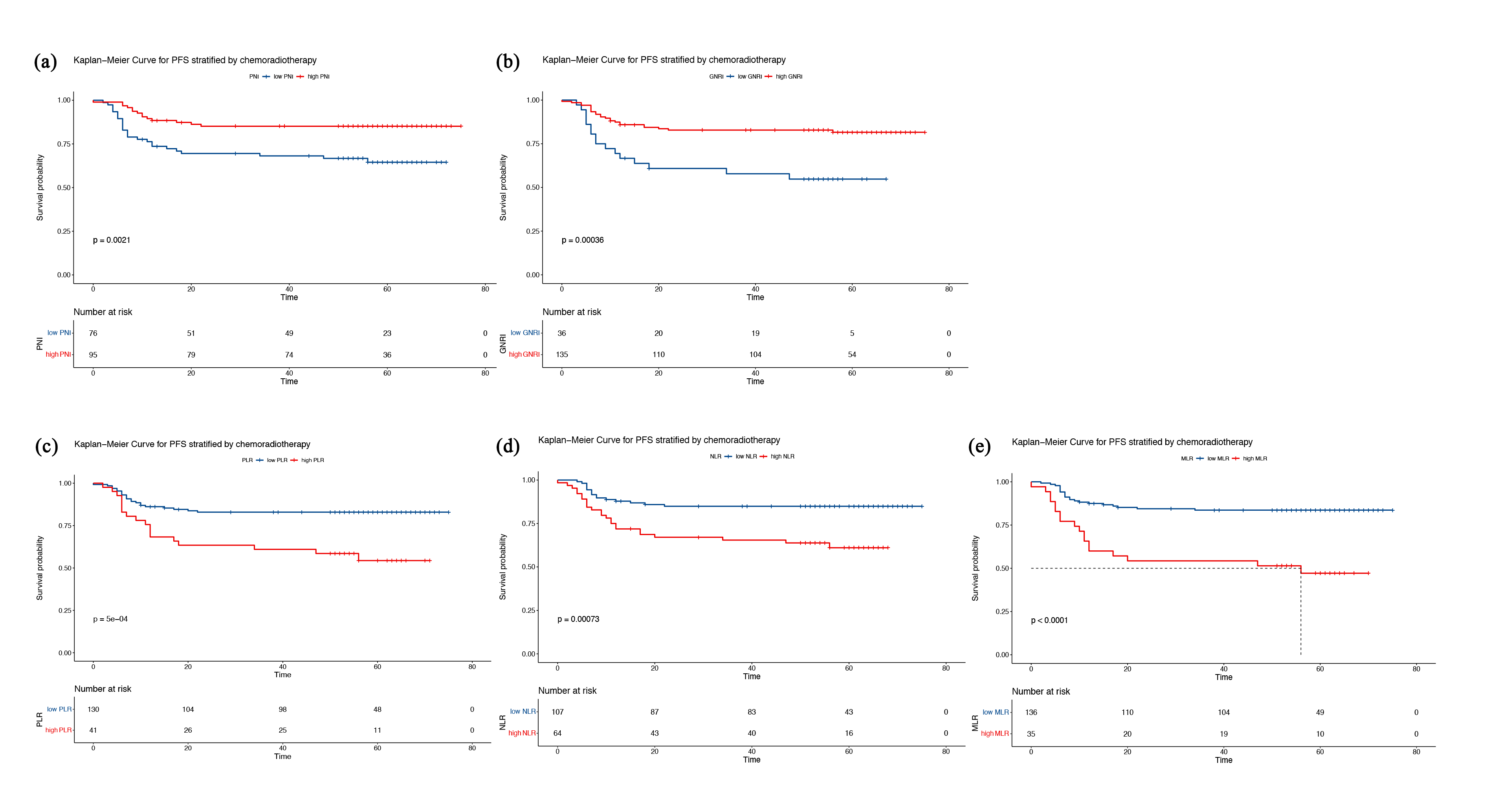


**Supplemental Figure 2.** Kaplan–Meier curves of progression-free survival stratified by chemoradiotherapy (CRT) according to nutritional and inflammatory indicators. (a) Low prognostic nutritional index (PNI) vs. high PNI (low PNI: ≤ 48.3, high PNI: > 48.3), (b) low geriatric nutritional risk index (GNRI) vs. high GNRI (low GNRI: ≤ 97.04, high GNRI: > 97.04), (c) low platelet/lymphocyte ratio (PLR) vs. high PLR (low PLR: ≤ 186.67, high PLR: > 186.67), (d) low neutrophil/lymphocyte ratio (NLR) vs. high NLR (low NLR: ≤ 2.8, high NLR: > 2.8), and (e) low monocyte/lymphocyte ratio (MLR) vs. high MLR (low MLR: ≤ 0.41, high MLR: > 0.41). The Kaplan–Meier method was used to calculate the survival rate, and the log-rank test was used to compare the survival distributions between the groups.


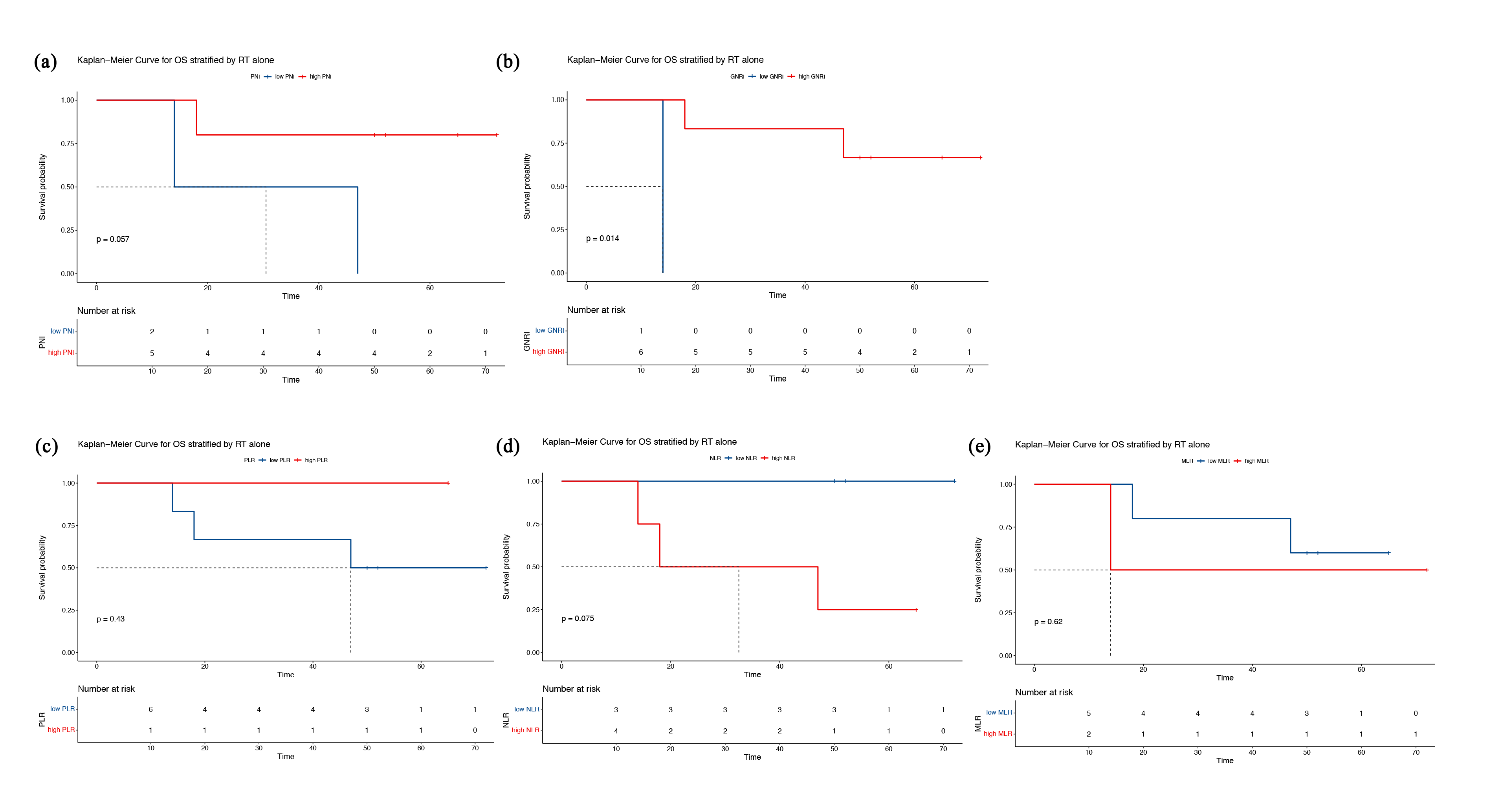


**Supplemental Figure 3.** Kaplan–Meier curves of overall survival stratified by radiotherapy (RT) alone according to nutritional and inflammatory indicators. (a) Low prognostic nutritional index (PNI) vs. high PNI (low PNI: ≤ 48.3, high PNI: > 48.3), (b) low geriatric nutritional risk index (GNRI) vs. high GNRI (low GNRI: ≤ 97.04, high GNRI: > 97.04), (c) low platelet/lymphocyte ratio (PLR) vs. high PLR (low PLR: ≤ 186.67, high PLR: > 186.67), (d) low neutrophil/lymphocyte ratio (NLR) vs. high NLR (low NLR: ≤ 2.8, high NLR: > 2.8), and (e) low monocyte/lymphocyte ratio (MLR) vs. high MLR (low MLR: ≤ 0.41, high MLR: > 0.41). The Kaplan–Meier method was used to calculate the survival rate, and the log-rank test was used to compare the survival distributions between the groups.


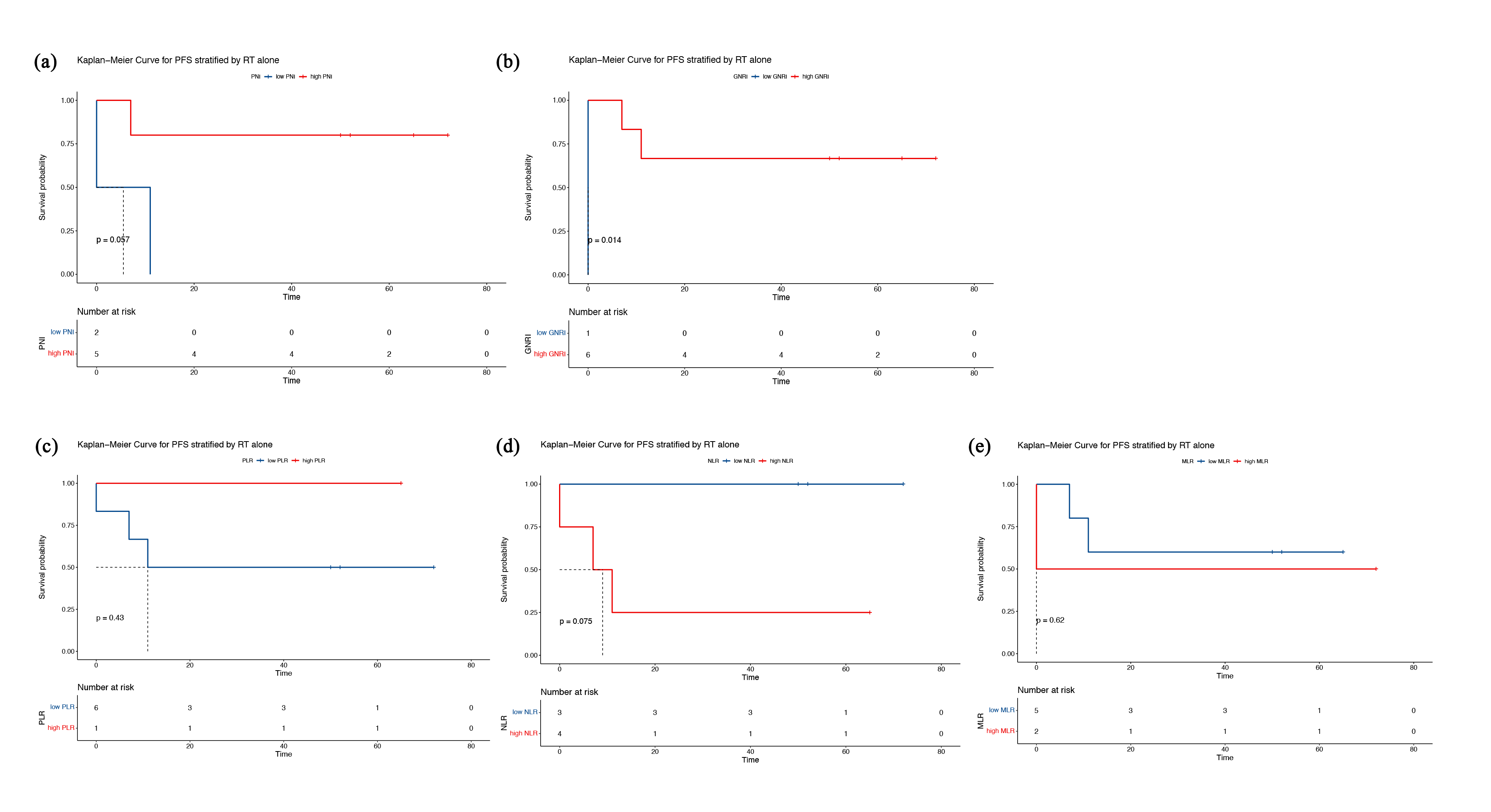


**Supplemental Figure 4.** Kaplan–Meier curves of progression-free survival stratified by radiotherapy (RT) alone according to nutritional and inflammatory indicators. (a) Low prognostic nutritional index (PNI) vs. high PNI (low PNI: ≤ 48.3, high PNI: > 48.3), (b) low geriatric nutritional risk index (GNRI) vs. high GNRI (low GNRI: ≤ 97.04, high GNRI: > 97.04), (c) low platelet/lymphocyte ratio (PLR) vs. high PLR (low PLR: ≤ 186.67, high PLR: > 186.67), (d) low neutrophil/lymphocyte ratio (NLR) vs. high NLR (low NLR: ≤ 2.8, high NLR: > 2.8), and (e) low monocyte/lymphocyte ratio (MLR) vs. high MLR (low MLR: ≤ 0.41, high MLR: > 0.41). The Kaplan–Meier method was used to calculate the survival rate, and the log-rank test was used to compare the survival distributions between the groups.

## Supplementary Tables

Supplementary Table 1 Univariate and multivariate analysis for overall survival stratified by chemoradiotherapy

| Variables | **Univariate analysis** | | **Multivariate analysis** | |
| --- | --- | --- | --- | --- |
|  | **HR (95%CI)** | ***P*-value** | **HR (95%CI)** | ***P*-value** |
| Age |  |  |  |  |
| ≤55 vs >55 | 0.61 (0.32-1.19) | 0.149 | 0.61 (0.30-1.23) | 0.166 |
| No. of metastatic lymph nodes |  |  |  |  |
| ≤2 vs >2 | 3.32 (1.75-6.32) | <0.001 | 1.92 (0.96-3.82) | 0.064 |
| Size of tumor |  |  |  |  |
| ≤4 cm vs >4 cm | 3.51 (1.72-7.19) | 0.001 | 2.53 (1.20-5.30) | 0.014 |
| Type of radiotherapy |  |  |  |  |
| IMRT vs RT | 1.16 (0.62-2.17) | 0.645 | - | - |
| Pathology |  |  |  |  |
| squamous cell carcinoma vs adenocarcinoma | 4.15 (1.97-8.76) | <0.001 | 4.08 (1.84-9.05) | 0.001 |
| FIGO Stage |  |  |  |  |
| II vs III | 3.94 (1.93-8.07) | <0.001 | 2.78 (1.33-5.83) | 0.007 |
| SCC |  |  |  |  |
| ≤ 1.5 vs > 1.5 | 1.24 (0.59-2.60) | 0.573 | - | - |
| PNI |  |  |  |  |
| ≤ 48.3 vs > 48.3 | 0.36 (0.19-0.69) | 0.002 | 0.53 (0.27~1.02) | 0.058 |
| GNRI |  |  |  |  |
| ≤ 97.04 vs > 97.04 | 0.31 (0.17-0.59) | <0.001 | 0.38 (0.19-0.77) | 0.007 |
| NLR |  |  |  |  |
| ≤ 2.8 vs > 2.8 | 2.97 (1.57-5.60) | 0.001 | 2.45 (1.26-4.76) | 0.008 |
| MLR |  |  |  |  |
| ≤ 0.41 vs > 0.41 | 4.05 (2.17-7.56) | <0.001 | 2.95 (1.49-5.85) | 0.002 |
| PLR |  |  |  |  |
| ≤ 186.67 vs > 186.67 | 3.03 (1.63-5.66) | <0.001 | 2.71 (1.37-5.37) | 0.004 |

Note: IMRT: Intensity-modulated radiotherapy; SCC: Squamous cell carcinoma; PNI: Prognostic nutritional index; GNRI: Geriatric nutritional risk index; NLR: Neutrophil/lymphocyte ratio; MLR: Monocyte/lymphocyte ratio; PLR: Platelet/lymphocyte ratio

Supplementary Table 2 Univariate and multivariate analysis for progression-free survival stratified by chemoradiotherapy

| Variables | **Univariate analysis** | | **Multivariate analysis** | |
| --- | --- | --- | --- | --- |
|  | **HR (95%CI)** | ***P*-value** | **HR (95%CI)** | ***P*-value** |
| Age |  |  |  |  |
| ≤55 vs >55 | 0.62 (0.32-1.20) | 0.156 | 0.52 (0.25-1.10) | 0.087 |
| No. of metastatic lymph nodes |  |  |  |  |
| ≤2 vs >2 | 3.27 (1.72-6.22) | <0.001 | 1.86 (0.94-3.69) | 0.076 |
| Size of tumor |  |  |  |  |
| ≤4 cm vs >4 cm | 3.45 (1.69-7.06) | 0.001 | 2.34 (1.12-4.90) | 0.024 |
| Type of radiotherapy |  |  |  |  |
| IMRT vs RT | 1.11 (0.59-2.08) | 0.740 | - | - |
| Pathology |  |  |  |  |
| squamous cell carcinoma vs adenocarcinoma | 4.92 (2.33-10.39) | <0.001 | 5.58 (2.43-12.83) | <0.001 |
| FIGO Stage |  |  |  |  |
| II vs III | 4.01 (1.96-8.21) | <0.001 | 3.02 (1.44-6.35) | 0.004 |
| SCC antigen |  |  |  |  |
| ≤ 1.5 vs > 1.5 | 1.19 (0.57-2.50) | 0.650 | - | - |
| PNI |  |  |  |  |
| ≤ 48.3 vs > 48.3 | 0.37 (0.20-0.72) | 0.003 | 0.57 (0.29-1.10) | 0.094 |
| GNRI |  |  |  |  |
| ≤ 97.04 vs > 97.04 | 0.33 (0.18-0.63) | 0.001 | 0.40 (0.20-0.79) | 0.009 |
| NLR |  |  |  |  |
| ≤ 2.8 vs > 2.8 | 2.83 (1.50-5.33) | 0.001 | 2.45 (1.25-4.80) | 0.009 |
| MLR |  |  |  |  |
| ≤ 0.41 vs > 0.41 | 3.90 (2.09-7.27) | <0.001 | 2.95 (1.49-5.85) | 0.002 |
| PLR |  |  |  |  |
| ≤ 186.67 vs > 186.67 | 2.89 (1.55-5.39) | 0.001 | 2.47 (1.25-4.88) | 0.009 |

Note: IMRT: Intensity-modulated radiotherapy; SCC: Squamous cell carcinoma; PNI: Prognostic nutritional index; GNRI: Geriatric nutritional risk index; NLR: Neutrophil/lymphocyte ratio; MLR: Monocyte/lymphocyte ratio; PLR: Platelet/lymphocyte ratio
